# *Lycium barbarum* Extracts Protect the Brain from Blood-Brain Barrier Disruption and Cerebral Edema in Experimental Stroke

**DOI:** 10.1371/journal.pone.0033596

**Published:** 2012-03-16

**Authors:** Di Yang, Suk-Yee Li, Chung-Man Yeung, Raymond Chuen-Chung Chang, Kwok-Fai So, David Wong, Amy C. Y. Lo

**Affiliations:** 1 Eye Institute, Li Ka Shing Faculty of Medicine, The University of Hong Kong, Hong Kong; 2 Department of Anatomy, Li Ka Shing Faculty of Medicine, The University of Hong Kong, Hong Kong; 3 Research Center of Heart, Brain, Hormone, and Healthy Aging, Li Ka Shing Faculty of Medicine, The University of Hong Kong, Hong Kong; Biological Research Centre of the Hungarian Academy of Sciences, Hungary

## Abstract

**Background and Purpose:**

Ischemic stroke is a destructive cerebrovascular disease and a leading cause of death. Yet, no ideal neuroprotective agents are available, leaving prevention an attractive alternative. The extracts from the fruits of *Lycium barbarum* (LBP), a Chinese anti-aging medicine and food supplement, showed neuroprotective function in the retina when given prophylactically. We aim to evaluate the protective effects of LBP pre-treatment in an experimental stroke model.

**Methods:**

C57BL/6N male mice were first fed with either vehicle (PBS) or LBP (1 or 10 mg/kg) daily for 7 days. Mice were then subjected to 2-hour transient middle cerebral artery occlusion (MCAO) by the intraluminal method followed by 22-hour reperfusion upon filament removal. Mice were evaluated for neurological deficits just before sacrifice. Brains were harvested for infarct size estimation, water content measurement, immunohistochemical analysis, and Western blot experiments. Evans blue (EB) extravasation was determined to assess blood-brain barrier (BBB) disruption after MCAO.

**Results:**

LBP pre-treatment significantly improved neurological deficits as well as decreased infarct size, hemispheric swelling, and water content. Fewer apoptotic cells were identified in LBP-treated brains by TUNEL assay. Reduced EB extravasation, fewer IgG-leaky vessels, and up-regulation of occludin expression were also observed in LBP-treated brains. Moreover, immunoreactivity for aquaporin-4 and glial fibrillary acidic protein were significantly decreased in LBP-treated brains.

**Conclusions:**

Seven-day oral LBP pre-treatment effectively improved neurological deficits, decreased infarct size and cerebral edema as well as protected the brain from BBB disruption, aquaporin-4 up-regulation, and glial activation. The present study suggests that LBP may be used as a prophylactic neuroprotectant in patients at high risk for ischemic stroke.

## Introduction

Ischemic stroke is a devastating cerebrovascular event and a leading cause of death worldwide. Stroke sufferers may present with various disabilities, including hemiplegia, dysesthesia, ataxia and even visual impairment. Two relevant mechanisms promoting these complications are extensive blood-brain barrier (BBB) disruption [1], [2] and cerebral edema [3], [4]. After ischemic onset, interruption of oxygen and glucose supply leads to cell death cascades which consequently result in BBB breakdown and cerebral edema [5]. Increased BBB permeability contributes to vasogenic edema, causing intravascular fluid to move to the surrounding brain parenchyma. This detrimental edema further reduces blood flow supplying the neurons, causing irreversible apoptosis [6], [7]. Extensive interactions among these three components become a vicious cycle, which accelerates brain damage.

Present management for acute ischemic stroke includes revascularization with thrombolytics and anti-edema therapy with surgical decompression or diuretics [8], [9], [10]. In order to salvage cell death, clinicians have also attempted to approach stroke patients with combining neuroprotective agents such as oxygen radical scavengers, NMDA receptor antagonists and MMP inhibitors [3], [11], [12], [13]; however, the outcomes are far less satisfactory. Given the devastative effects and social burden of stroke, preventive measures may be one of the many strategies in stroke management.

Recent studies have reported that pre-administration of neuroprotective agents are beneficial in experimental stroke models. Adrenomedullin, rennin inhibitor aliskiren and Hawthorn extract when given prophylactically showed protective effects in suppressing cerebral edema and cell apoptosis as well as improving neurological outcome [14], [15], [16]. In the present study, the extracts from the fruit of *Lycium barbarum* (also named Wolfberry, *Fructus Lycii*, Gouqizi) which has been widely used in Chinese herbal recipes for thousands of years was tested for its neuroprotective effects in ischemic stroke.

*Lycium barbarum* is an important ingredient in traditional Chinese medicine in promoting health and longevity as well as a food supplement in the Western countries. Valuable components of *Lycium barbarum* are not limited to its colored components containing lutein and zeaxanthin, but include the polysaccharides which constitute more than 40% of the fruit extract [17]. It has been shown that pre-treatment using extracts of *Lycium barbarum* containing mostly polysaccharides (LBP) could protect the cultured primary cortical neurons from β-amyloid peptide neurotoxicity [18], [19]. We also found that LBP pre-treatment could effectively protect the retina from neuronal death, glial activation and oxidative stress in a murine retinal ischemia/reperfusion model [20]. Taken together, we hypothesize that LBP pre-treatment may be neuroprotective in ischemic stroke. In this study, we evaluated the protective effects of LBP in a murine transient cerebral ischemia/reperfusion model and further showed that LBP pre-treatment could reduce cerebral edema and BBB disruption in the early stage of stroke.

## Materials and Methods

### Ethics Statement

The use of animals in this study was conducted according to the requirements of the Cap. 340 Animals (Control of Experiments) Ordinance and Regulations, and all relevant legislation and Codes of Practice in Hong Kong. All the experimental and animal handling procedures were approved by the Faculty Committee on the Use of Live Animals in Teaching and Research in The University of Hong Kong (CULATR #1870-09).

### Animals

C57BL/6N male mice (10–12 weeks old) used in the present study were housed under diurnal lighting condition and allowed free access to food and water.

### Transient Cerebral Ischemia

Transient cerebral ischemia was induced by surgical occlusion of the middle cerebral artery. The procedures of middle cerebral artery occlusion (MCAO) were performed as previously described [10], [21]. Briefly, the mouse was anesthetized (induction with 2% halothane in 70% nitrous oxide and 30% oxygen; maintenance with 1% halothane in 70% nitrous oxide and 30% oxygen) through a facemask [10], [21]. An 8-0 filament coated with vinyl polysiloxane (3M Dental Products, St. Paul, MN, USA) was inserted into the right internal carotid artery to occlude right middle cerebral artery at its origin. To confirm successful obstruction and reperfusion of the artery, relative cerebral blood flow (CBF) in the right middle cerebral artery core territory was monitored through an optic fiber glued to the skull (2 mm posterior and 6 mm lateral to bregma) and connected to a laser Doppler flowmeter (Perimed, Järfälla, Sweden). After filament insertion, the gas anesthesia was continued for another 5 minutes to ensure maintenance of ischemia. Then, the anesthesia was removed and the animal was transferred to an incubator (ThermoCare® intensive care unit system, ThermoCare Inc, Incline Village, NV, USA) at 30°C. At 10 minutes before reperfusion (i.e. 1 hour and 50 minutes after ischemia), the animal was anesthetized again as before and the filament was removed at 2 hours after ischemia to allow reperfusion. The anesthesia was continued for another 5 minutes after which the animal was kept in an incubator for 4 hours. During the experiment, the rectal temperature was kept by the heating pad at about 37±1.5°C.

### *Lycium barbarum* Polysaccharides Preparation and Drug Treatment

*Lycium barbarum* polysaccharides (LBP) extracts were prepared as previously described [19], [22]. Briefly, the dried fruit of *Lycium barbarum* (10 kg) were pulverized into small particles and saturated in 95% ethanol. Subsequently, the remaining residue was filtered and air-dried. The dry *Lycium barbarum* residue was then dissolved in 70°C water, and the supernatant of which was concentrated, precipitated with 95% ethanol and then vacuum dried to produce the LBP extracts (2 g).

For treatment, LBP extracts were dissolved in phosphate-buffered saline (PBS; 0.01M; pH 7.4). Mice were randomly divided into 3 groups: PBS-treated group (Vehicle), 1 mg/kg LBP-treated group (LBP1) and 10 mg/kg LBP-treated group (LBP10). These dosages were chosen based on our previous *in vivo* studies [22]. Animals were fed with LBP or PBS daily through a gastric tube for 7 days prior to the induction of MCAO as Chinese usually drink the soup containing *Lycium barbarum* for several days [22].

### Evaluation of Neurological Deficits, Infarct Size, and Hemispheric Brain Swelling

Mice were evaluated for neurological deficits 22 hours after reperfusion as previously described [10], [21]. Briefly, mice were scored in a double-blinded manner as follows: 0, no observable neurological deficits (normal); 1, failure to extend opposite forepaw (mild); 2, circling to the contralateral side (moderate); and 3, loss of walking and righting reflex (severe). Mice were sacrificed immediately after scoring. Brains were cut into 6 coronal slices in 2-mm thickness, stained with 2% 2,3,5-triphenyltetrazolium chloride (TTC) (Sigma, St. Louis, MO, USA) at 37°C in dark to detect ischemic area and subsequently fixed in 10% buffered formalin overnight. The posterior surface of each brain slice was photographed and analyzed using a digital image analysis system (SigmaScan Pro, SPSS, Chicago, IL, USA) for the determination of infarct area, infarct volume and hemispheric swelling. Infarct area and volume were estimated using the indirect method [10], [21]. Hemispheric swelling was determined as 100%×(ipsilateral volume−contralateral volume)/contralateral volume [8], [10], [21].

### Terminal Deoxynucleotidyl Transferase Biotin-dUTP Nick-End Labeling (TUNEL)

Apoptosis in brain sections was investigated by TUNEL assay (DeadEnd Fluorometric TUNEL system, Promega, Madison, WI) as previously described [23]. Sections were counterstained with 4′,6-diamidino-2-phenylindole (DAPI) after the reaction to validate the nuclear location of TUNEL-positive signal. Quantification of TUNEL-positive cells was analyzed along ipsilateral ischemic penumbra area. At least ten microscopic fields (40× magnification) were counted in a double-blinded manner and averaged in each section [24].

### Tissue Processing and Immunohistochemical Analyses

Fixed brain slices were dehydrated and embedded in paraffin. For immunohistochemical (IHC) analyses, 7-µm paraffin brain sections were deparaffinized, rehydrated and blocked with normal goat serum (mouse on mouse blocking solution for IgG). Sections were then incubated with antibodies against IgG (1∶200, MOM kit, Vector Laboratories, Burlingame, CA, USA), occludin (1∶50, Zymed, CA, USA), aquaporin-4 (AQP4 1∶500, Millipore, MA, USA), glial fibrillary acidic protein (GFAP 1∶500, Dako, Denmark), nitrotyrosine (NT 1∶200, Upstate Biotechnology, NY, USA), poly (ADP-ribose) (PAR 1∶200, Alexis, Lausen, Switzerland) and matrix metalloproteinase-9 (MMP-9 1∶500, Abcam, MA, USA) overnight at 4°C. Subsequently, brain sections were incubated with corresponding secondary antibody (1∶200, Vector Laboratories, Burlingame, CA, USA) for 45 minutes at room temperature. Diaminobenzadine (DAB) (Zymed, CA, USA) staining was performed by incubating the sections with 0.9% hydrogen peroxide and Vectastain ABC reagent (Vector Laboratories, Burlingame, CA, USA) for 30 minutes at room temperature. The immunoreactivity was visualized with DAB staining and sections were counterstained with hematoxylin. Slides were analyzed and scored according to the immunoreactivity intensity and distribution as previously described [20], [25]. Score 5 and score 1 indicated the highest immunoreactivity and the weakest immunoreactivity, respectively. All procedures were carried out in a double-blinded manner.

### Water Content Measurement

A separate group of mice (n = 7) were used to evaluate the water content. Mouse brains were harvested at 22 hours after reperfusion for determination of water content in the cerebral tissue. Wet and dry weight of cerebral hemispheres (ipsilateral and contralateral) was measured and the corresponding water content was calculated by the formula water content = 100% (wet weight−dry weight)/wet weight [1], [26].

### Blood-Brain Barrier (BBB) Permeability

Another group of mice (n = 5) were operated to investigate the integrity of BBB. BBB permeability was assessed by measuring extravasated Evans blue (EB) dye. 0.1 ml of 4% EB (Sigma, USA) in saline was injected through tail vein right after reperfusion. Mice were sacrificed 22 hours after reperfusion by transcardial perfusion of heparinized saline. The amount of EB in the cerebral hemispheres was quantified at 630 nm by spectrofluorophotometry as previously described [1], [2]

### Western Blot Analysis

A new group of mice were used to analyze the expression of occludin. Proteins were collected from ipsilateral hemispheres of different groups as previously described with slight modification [10]. Briefly, ipsilateral brain slice 3 and 4 were homogenized in RIPA lysis buffer [20] and protein concentrations were measured by protein assay (Bio-Rad, USA). Blots were blocked with 5% milk for 1 hour at room temperature. Subsequently, blots were incubated with antibody against occludin (1∶500, Zymed, CA, USA) overnight at 4°C and probed with the corresponding secondary antibody (1∶2000, Vector Laboratories, Burlingame, CA, USA) for 1 hour at room temperature. Signals were quantified by ImageJ (NIH, USA) and normalized against actin. All procedures were carried out in a double-blinded manner.

### Statistical Analysis

Data were presented as mean ± SEM. Statistical analysis was performed by one-way ANOVA with Bonferroni multiple comparison tests in quantitative analysis and Kruskal-Wallis Test followed by Dunn's multiple comparison tests in qualitative analysis (GraphPad Prism software, San Diego, CA, USA). Statistical significance was assumed when *P*<0.05.

## Results

### Similar Relative CBF and Rectal Temperature

During experiments, the relative CBF in the right middle cerebral artery core territory was monitored by laser Doppler flowmeter to ensure successful occlusion and reperfusion of the right middle cerebral artery. The rectal temperature was kept by the heating pad at 37±1.5°C. Our results indicated that mice in the three experimental groups had very similar relative CBF and rectal temperature during ischemia and reperfusion (Table 1), suggesting that all the animals were subjected to similar degree of ischemic insult and rectal temperature.

**Table 1:** Relative cerebral blood flow and body temperature of the mice during ischemia and after reperfusion.

	Relative cerebral blood flow (%)(Rectal temperature °C)			
	5 min before ischemia	5 min after ischemia	5 min before reperfusion	5 min after reperfusion
**Vehicle (n = 8)**	100(36.9±0.2)	22.4±2.3 (37.1±0.1)	20.1±2.5(38.5±1.2)	165.2±21.2(37.3±0.5)
**LBP 1 (n = 7)**	100(36.8±0.2)	22.2±4.4(37.0±0.2)	21.9±3.6(38.5±1.2)	126.8±18.6(37.2±0.7)
**LBP 10 (n = 7)**	100(37.0±0.1)	24.4±3.1(37.2±0.1)	23.2±3.2(38.4±0.6)	143.9±31.5(37.2±0.3)

### Improved Neurological Deficits

Mice were evaluated for neurological deficits at 22 hours after reperfusion just before sacrifice. We observed more severe neurological deficits in the vehicle group, while the neurological scores were significantly improved by 10 mg/kg LBP pre-treatment (*P*<0.05, Table 2).

**Table 2:** Less severe neurological deficits in LBP-treated mice after MCAO.

	Distribution of neurological scores				
Score	0	1	2	3	Mean±SEM
**Vehicle (n = 8)**	0	2	4	2	2.00±0.27
**LBP 1 (n = 7)**	0	4	3	0	1.43±0.20
**LBP 10 (n = 7)**	0	6	1	0	1.14±0.14*

* P<0.05 LBP10 vs. vehicle, Kruskal-Wallis test followed by Dunn's Multiple Comparison Test.

### Decreased Cerebral Infarct Size and Hemispheric Swelling

**Figure 1:**
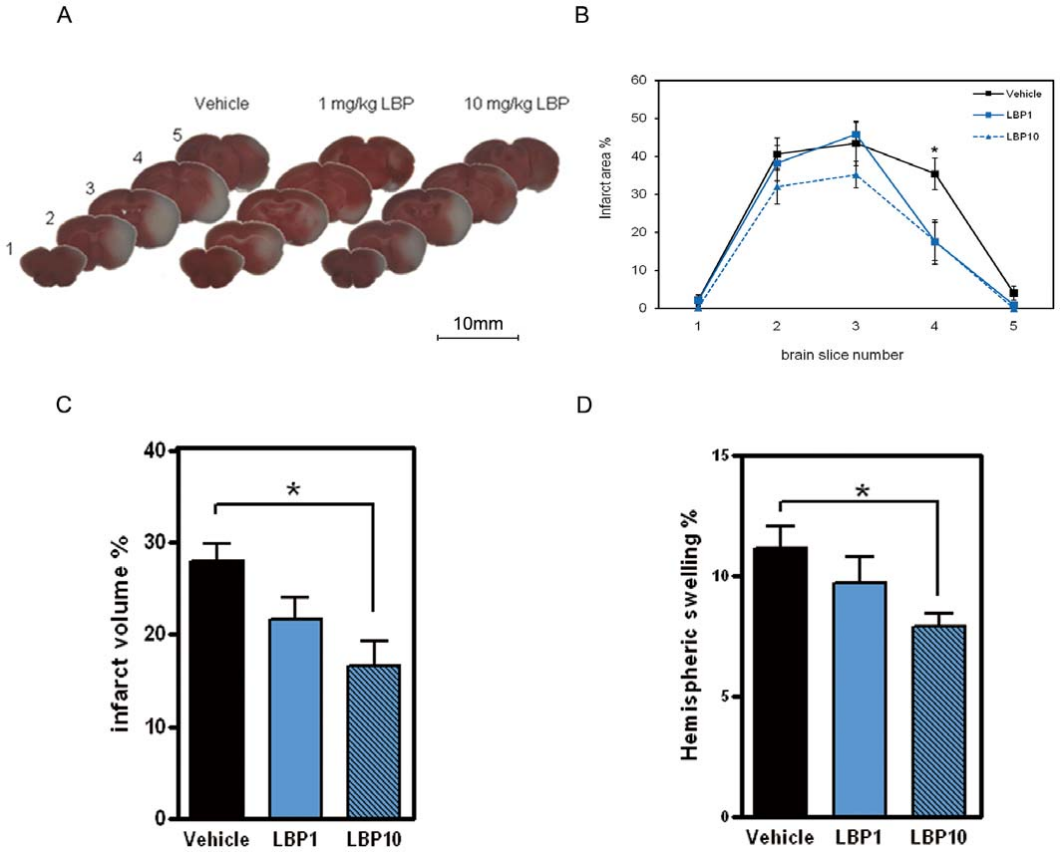
Decreased infarct size and hemispheric swelling in LBP-treated brains after MCAO. (A) Representative photographs of coronal brain slices stained with 2% TTC. Slice 1, most rostral; slice 5, most caudal. Note the smaller white regions indicating reduced infarct areas in LBP-treated brains. Scale bar = 10 mm. LBP-treated brains showed significantly decreased infarct area % (B), infarct volume % (C) and hemispheric swelling % (D) when compared with vehicle-treated brains. *P<0.05, ANOVA followed by Bonferroni's test, n = 7 to 8 for all groups.

TTC-staining results showed that the infarct area of brain slice number 4 significantly dropped from about 36% in vehicle-treated brains to approximately18% in both LBP groups (Figure 1B). Moreover, the total infarct volume was also reduced in the mice treated with LBP, especially with 10 mg/kg pre-treatment (*P*<0.05 versus vehicle group, Figure 1C). Consistent with the decreased infarct size, less hemispheric swelling was also observed in ipsilateral cerebrum of LBP-treated mice (Figure 1D).

### Fewer Apoptotic Cells in Ischemic Penumbra Area

**Figure 2:**
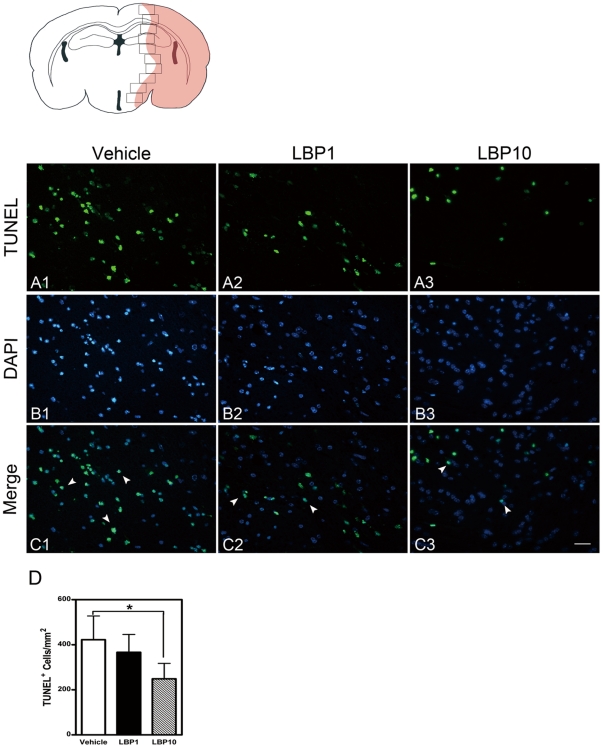
Fewer apoptotic cells in LBP group. The upper-left schematic diagram showed the area where TUNEL assay was analyzed (interaural 1.98 mm). The red shadow indicated the infarct area after MCAO. TUNEL-positive cells were counted in penumbral areas (black frames). (A) TUNEL. (B) DAPI. (C) Merged images of TUNEL and DAPI (arrow heads). In LBP-treated groups, fewer TUNEL-positive cells were observed (C2 & C3) when compared with vehicle group (C1). Scale bars = 25 µm. (D) Quantification of TUNEL-positive cells showing the decrease of apoptosis in LBP-treated brains. * *P*<0.05, ANOVA followed by Bonferroni's test, n = 5 each group.

Two-hour ischemia led to irreversible death of cerebral neurons. Numerous TUNEL-positive cells were observed in vehicle-treated brains in ischemic penumbral area. Yet, we found only a few in LBP-treated brains (Figure 2). Quantification of TUNEL-positive cells showed a significant decline with pre-treatment of LBP (249.0 cells/mm^2^ in LBP10 group vs. 422.2 cells/mm^2^ in vehicle group, *P*<0.05).

### Less Brain Water Content

**Figure 3:**
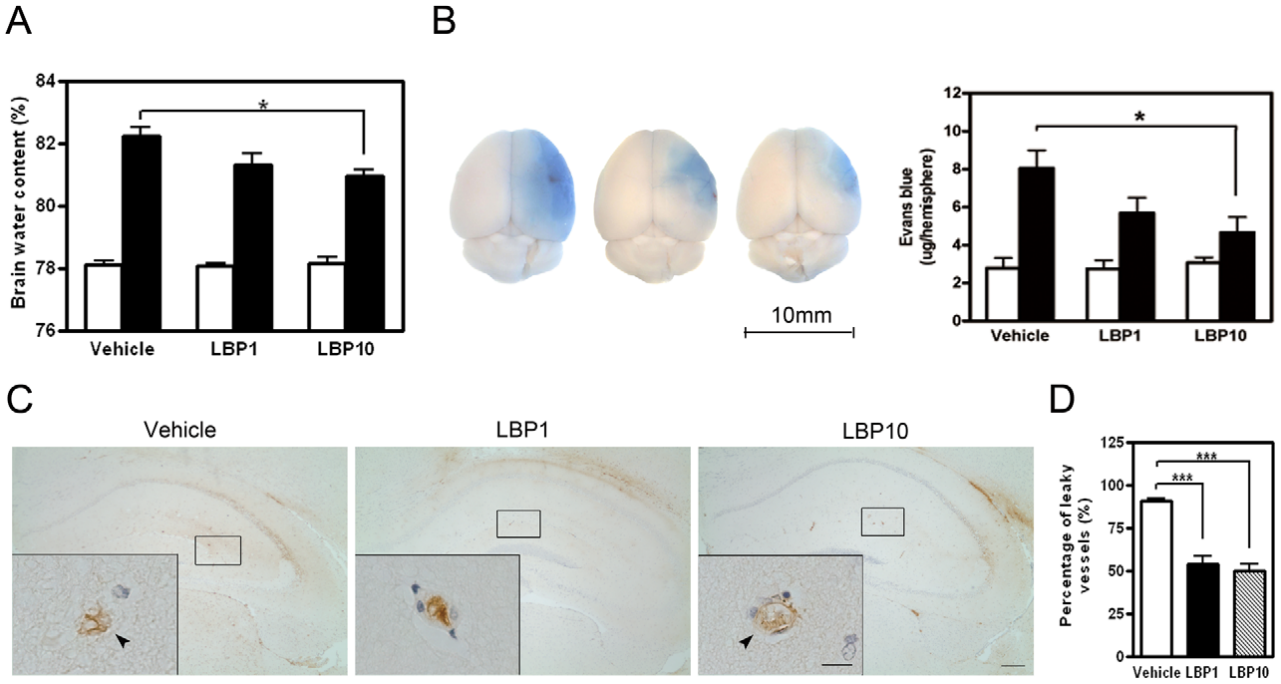
Reduced water content and blood-brain barrier (BBB) disruption in LBP-treated cerebral hemispheres. (A) Water content in vehicle and LBP-treated cerebral hemispheres 22 hours after reperfusion. White bars, contralateral hemisphere; filled bars, ipsilateral hemisphere. *P<0.05, ANOVA followed by Bonferroni's test, n = 7 each group. (B) Representative photographs of brains after Evans blue (EB) extravasation assay (left). Scale bar = 10 mm. Leakage of EB (blue area) indicated BBB breakdown after MCAO. LBP-treated ipsilateral hemisphere showed decreased EB extravasation (right). White bars, contralateral hemisphere; filled bars, ipsilateral hemisphere. *P<0.05, ANOVA followed by Bonferroni's test, n = 5 each group. (C) Representative IgG IHC showing leaky blood vessels in ipsilateral penumbral areas (interaural 1.98 mm). IgG signal leaked outside the blood vessel lumen (arrow head) in vehicle-treated brain. In LBP-treated brains, the outline of blood vessels was mostly intact and the IgG signal was present inside the vessel lumen. Inserts, higher magnification of typical blood vessels. Scale bar = 200 µm, inserts scale bar = 25 µm. (D) Quantification of blood vessel leakage in ipsilateral penumbral areas. Fewer leaky vessels were observed in LBP-treated brain when compared with the vehicle group. ***P<0.001, ANOVA followed by Bonferroni's test, n = 5 each group.

Decreased water content was found in ipsilateral hemispheres of LBP-treated mice, which indicated less cerebral edema (*P*<0.05 LBP10 group vs. vehicle group, Figure 3A). Water content of the contralateral hemispheres in all three groups was similar.

### Alleviated Blood-Brain Barrier Breakdown after Transient MCAO

Evans Blue dye leaked mainly into the ipsilateral hemispheres in all experimental groups when compared with the minimal amount in the contralateral side. LBP pre-treatment markedly decreased the ipsilateral EB leakage, indicating that LBP reduced the BBB permeability after transient MCAO (Figure 3B). This was further confirmed by immunohistochemical analysis of IgG localization. In vehicle-treated brains, the outline of blood vessels was discontinuous and IgG immunoreactivity leaked outside the vessel lumen, suggesting an elevated BBB permeability after MCAO (Figure 3C). In line with the EB results, a smaller percentage of leaky vessels was present in LBP-treated brains showing less BBB disruption (*P*<0.001 versus vehicle group, Figure 3D).

**Figure 4:**
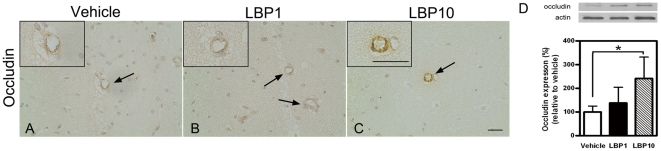
Stronger expression of occludin. (A–C) Representative IHC showing occludin signal in blood vessels in ipsilateral penumbral areas (interaural 1.98 mm). Occludin immunoreactivity was stronger in LBP10 group (arrow, C). Inserts, higher magnification of typical blood vessels. Scale bar = 25 µm, inserts scale bar = 25 µm. (D) Western blots exhibiting higher level of occludin in LBP group. *P<0.05, ANOVA followed by Bonferroni's test, n = 3–5 for all groups.

Occludin is one of the proteins located at tight junctions which played an important role in integrity of BBB. Previously, a decreased level of occludin in ischemic brains after MCAO was observed by Western blot [10], [27]. The current Western blots analyses revealed an up-regulation of occludin expression in both LBP1 and LBP10 groups, supporting alleviated BBB breakdown (*P*<0.05 LBP10 group versus vehicle group, Figure 4D). In addition, LBP pre-treatment enhanced the immunoreactivity of occludin along the outline of blood vessels (Figure 4A–C).

### Down Regulation of Aquaporin-4 Expression

**Figure 5:**
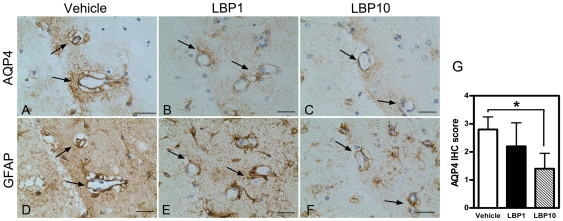
Decreased immunoreactivity of AQP4 in LBP-treated brain. (A–C) AQP4 IHC signals in swollen end-feet of astrocytes around cerebral vessels in ipsilateral penumbral areas (interaural 1.98 mm). Note the intense AQP4 staining in vehicle-treated vessels after MCAO (arrows, A), which was decreased in both LBP groups (B&C). (D–F) GFAP IHC using adjacent section to AQP4 IHC. Note the GFAP immunoreactivity located around the same cerebral vessels as in the AQP4 immunoreactivity (arrows). Scale bar = 25 µm. (G) Semi-quantification of immunoreactivity of AQP4. *P<0.05, Kruskal-Wallis followed by Dunn's multiple comparison test, n = 5 each group.

AQP4, a protein responsible for water transport in the brain, was up-regulated by cerebral ischemic insult especially in the astrocytic end-feet adjacent to the blood vessels [28]. However, LBP pre-treatment profoundly suppressed the up-regulation of AQP4 expression (Figure 5B, C). To semi-quantify the AQP4 expression, IHC scoring was performed. LBP10 group received a score half of that in the vehicle group, verifying the reduction in AQP4 up-regulation (*P*<0.05 LBP10 group versus vehicle group, Figure 5G).

### Lower Glial Fibrillary Acidic Protein Activation

**Figure 6:**
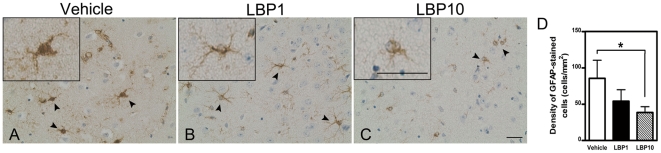
Fewer activated glial cells in LBP-treated brain. (A–C) Representative IHC showing activation of GFAP in ipsilateral penumbral areas after MCAO (interaural 1.98 mm). Arrow heads, typical GFAP-positive astrocytes. Fewer GFAP-positive astrocytes were observed in LBP-treated brains (B & C). Inserts, higher magnification of GFAP-positive astrocytes. Scale bar = 25 µm, inserts scale bar = 25 µm. (D) Quantification of GFAP-stained cells. *P<0.05, ANOVA followed by Bonferroni's test, n = 4–5 for all groups.

GFAP-stained astrocytes showed highly activated swollen processes in ipsilateral penumbral area after MCAO (Figure 6A). However, this activation was markedly reduced in LBP groups (Figure 6B, C). Moreover, quantitative analysis showed that the density of GFAP-positive cells was decreased to 38.6 cells/mm^2^ in LBP10 group when compared with 85.6 cells/mm^2^ in the vehicle group (*P*<0.05, Figure 6D).

### Reduced Oxidative Stress

**Figure 7:**
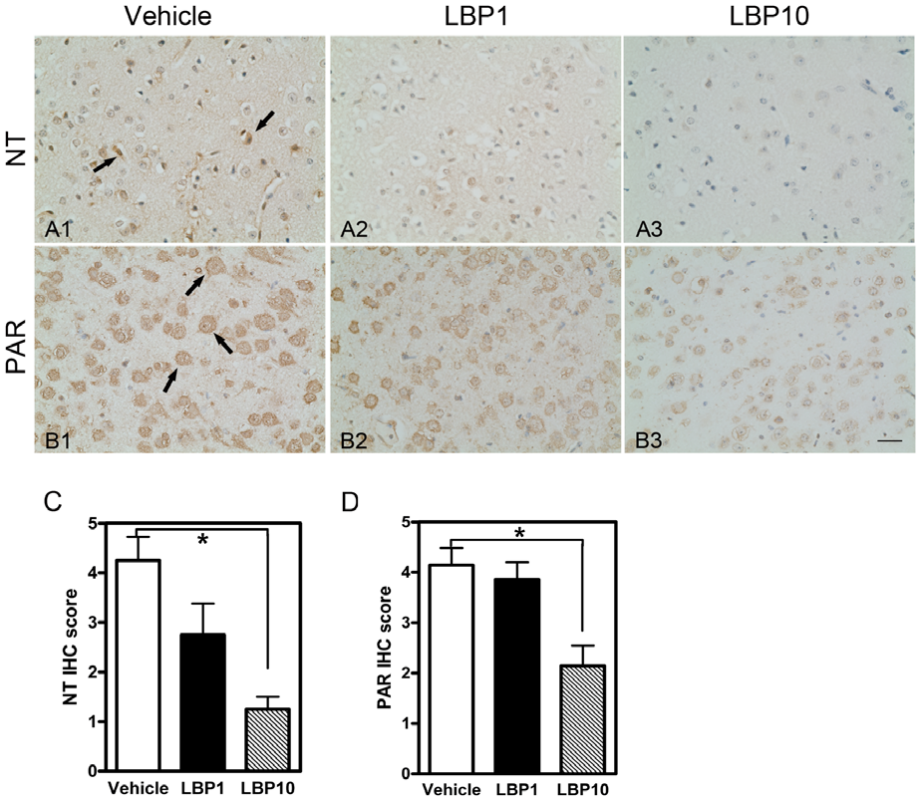
LBP pre-treatment reduced both nitrosative stress and lipid peroxidation in cerebral ischemic penumbra after MCAO. (A1–A3) Immunohistochemistry of nitrotyrosine (NT). Increased immunoreactivity of NT was noted in vehicle-treated brains after MCAO (arrows, A1). However, minimal NT signal was found in LBP groups (A2 & A3). (B1–B3) Immunohistochemistry of poly(ADP-ribose) (PAR). A profound PAR expression was observed in vehicle-treated brains (arrows, B1). With LBP treatment (LBP10), PAR immunoreactivity was much reduced (B3). (C & D) Semi-quantification of IHC (C, NT; D, PAR). * *P*<0.05, Kruskal-Wallis followed by Dunn's multiple comparison test, n = 5–7 for all groups. Scale bars = 25 µm.

Nitrotyrosine (NT) is considered as a marker of nitrosative stress. Increased immunoreactivity of NT was observed in vehicle-treated brain (Figure 7A1), while LBP groups exhibited minimal NT signal (Figure 7A2, A3). More importantly, the IHC score in LBP10 group was below half of that in the vehicle-treated group, verifying the reduction in NT expression (*P*<0.05, Figure 7C).

Poly (ADP-ribose) (PAR), activated by lipid peroxidation under ischemic conditions, is also a marker of oxidative stress. A profound PAR expression was noted in vehicle-treated brain (Figure 7B1), suggesting an increased level of lipid peroxidation after MCAO. LBP pre-treatment at 10 mg/kg markedly suppressed the immunoreactivity of PAR (Figure 7B3). To semi-quantify the PAR expression, IHC scoring was performed. LBP10 group received a score half of that in the vehicle group, confirming the decline in lipid peroxidation (*P*<0.05 LBP10 group versus vehicle group, Figure 7D).

### Attenuated Expression of Matrix Metalloproteinase-9

**Figure 8:**
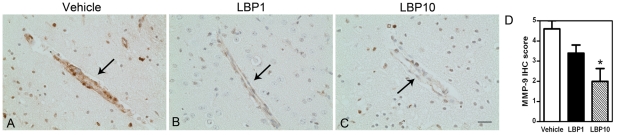
Decreased immunoreactivity of MMP-9 in LBP-treated brain. (A–C) MMP-9 IHC signals in endothelial cells along vessels in ipsilateral penumbral areas (interaural 1.98 mm). Note the intense MMP-9 staining in vehicle-treated vessels after MCAO (arrow, A), which was reduced in both LBP groups (arrows, B&C). Scale bar = 25 µm. (D) Semi-quantification of immunoreactivity of MMP-9. *P<0.05, LBP10 vs. Vehicle, Kruskal-Wallis followed by Dunn's multiple comparison test, n = 5 each group.

MMP-9 immunoreactivity was significantly elevated after MCAO in vehicle-treated brain (Figure 8A). Intense MMP-9 expression appeared mostly in endothelial cells in ipsilateral penumbral areas. However, an attenuated level of MMP-9 immunoreactivity was noted in both LBP1 and LBP10 group (Figure 8B, C) indicating that LBP could suppress the up-regulation of MMP-9 after cerebral ischemia. Moreover, the IHC score in vehicle, LBP1 and LBP10 were 4.6±0.4, 3.4±0.4 and 2.0±0.6 arbitrary units, respectively (*P*<0.05, Figure 8D).

## Discussion

The fruit of *Lycium barbarum* (Gouqizi, Wolfberry, *Fructus Lycii*) has been widely used as a traditional medicinal ingredient and food supplement in China for decades. Also, it has become increasingly popular in western countries as an anti-aging remedy [29]. So far, no side effects or toxicity have been reported [29]. Recent investigations of *Lycium barbarum* have focused on its valuable components, known as *Lycium barbarum* polysaccharides (LBP), which constitutes more than 40% of the fruit extract. Major components in LBP include glucose, arabinose, galacturonic acid and galactose [19]. We showed in this study that LBP extracts exerted significant neuroprotection when given prophylactically. This may be one beneficial effect for patients at high risk for ischemic stroke.

Besides LBP, other polysaccharide extracts have also been shown to possess neuroprotective effects in cerebral ischemic injuries. For instance, polysaccharides from *Hyriopsis cumingii* exhibited neuroprotective functions through anti-apoptotic and anti-oxidative activities in rat cerebral ischemia/reperfusion model [30]; *Ganoderma lucidum* polysaccharides could protect against rat cerebral ischemia by inhibiting apoptosis and the potential mechanisms may be associated with the modulation of Bcl-2 and caspase-3 pathway [31]; Cactus polysaccharides could reduce neuronal apoptosis and oxidative stress in rat cerebral ischemia as well [32]. Here, we report for the first time that LBP could alleviate BBB disruption and cerebral edema resulting in a significant neuroprotection after ischemic insult.

Acute excitotoxicity, oxidative stress and inflammation are three primary mechanisms involved in cell death during ischemic stroke. They markedly damage the neurovascular unit, which includes neurons, glia and vascular components. [9]. Cell death within infarct core might be irreversible without early reperfusion; however, promising neuroprotective agents which could prevent the neuronal damage bring hope to salvage the dying cells in the penumbral area. Our previous study showed that pre-treatment of LBP could protect the retinal ganglion cells from apoptosis in a retinal ischemia model [20]. In the present investigation, improved neurological deficits and smaller infarct size were observed in LBP-treated mice. Moreover, we noted fewer apoptotic cells in the penumbral area by TUNEL assay which was similar to our findings in retina. Further observations on nitrosative stress and lipid peroxidation (NT and PAR) suggested that the anti-oxidative effects of LBP might contribute to smaller infarction and better neurological outcome. We have also validated the neuroprotective effects of LBP pre-treatment on cultured neurons and speculated that the possible mechanism may be its inhibitory effects in JNK and ERK signaling pathway [18], [33].

Cerebral ischemia and reperfusion triggers a cascade of cellular events including cell death, oxidative stress and inflammation which all contribute to the breakdown of BBB [9]. BBB disruption further aggravates either vasogenic edema or hemorrhagic transformation which leads to severe neurological deficits [1]. In our study, we investigated the integrity of BBB by Evans blue assay and IgG immunohistochemistry which both revealed a decreased permeability of BBB after LBP pre-treatment. Furthermore, less disrupted tight junctions indicated by higher expression of occludin in LBP-treated brains supported the current findings. These results were consistent with our previous data which showed LBP pre-treatment could protect the blood-retinal barrier disruption after retinal ischemia/reperfusion injury [20]. It is widely accepted that matrix metalloproteinases (MMP) are major enzymes positively associated with disruption of BBB after ischemic injuries. Elevated MMP-9 expression was observed within the first three days after ischemic stroke and it exhibited close connection with the extent of BBB breakdown [34]. In line with previous studies [35], [36], an increased level of MMP-9 immunoreactivity was noted in vehicle-treated brains at 24 hours after cerebral ischemia in our investigation. As expected, MMP-9 expression was mainly located in the endothelial cells along the vessels in penumbral areas. With 7-day LBP pre-treatment, the elevated expression of MMP-9 was markedly attenuated, indicating that the protective effects of LBP on BBB disruption might partially relate to the down-regulation of MMP-9.

Cerebral edema is a detrimental feature after ischemic stroke and one of the impact factors of clinical deterioration within the first 24 hours after stroke onset [37]. Swelling caused by edema brings harmful effects on adjacent non-ischemic tissues due to the fixed volume of the skull and further affects the prognosis of patients [4], [38]. Here, we observed that pre-treatment of LBP reduced cerebral swelling and water content in the injured hemisphere, suggesting that LBP is helpful to lessen cerebral edema after MCAO. Apart from these findings, we further investigated the effects of LBP on AQP4. AQP4 is a principal water-channel protein responsible for water movement in the brain parenchyma. Multiple studies have shown that the expression of AQP4 is up-regulated after cerebral ischemic injuries [10], [28], [39]. Lately, AQP4 has become a potential therapeutic target to treat cerebral edema during ischemic stroke [39]. Our study revealed lower immunoreactivity of AQP4 in LBP-treated brains, illustrating that the anti-edema effects of LBP may partly act through its modulation on AQP4 expression.

It should be noted that the relative CBF measurement in this study indicated only the blood flow data obtained from a spot at the center of the middle cerebral artery territory i.e. ischemic core. In fact, there is a possibility that blood flow in the penumbra is altered by LBP pre-treatment. However, at this point, there is not enough evidence in the current study to show that blood flow in the penumbra is altered by LBP pre-treatment. Further investigation is necessary to explore the effects of LBP in improving cerebral blood flow after stroke.

Taken together, the neuroprotective effects of LBP pre-treatment on ischemic stroke include anti-apoptosis, conservation of BBB integrity and alleviation of cerebral edema. Based on the current results, these protective effects might act through anti-oxidation and down-regulation of MMP-9. The present study clearly suggests the beneficial prophylactic effects of LBP against ischemic damage and cerebral edema in a murine experimental stroke model. Although the active ingredients of LBP in protecting ischemic stroke remains unknown, LBP pre-stroke dosing regimen may have the potential in translating into clinical situations for high-risk patients.
